# Quality of Life in Chinese Patients With Large Congenital Melanocytic Nevi

**DOI:** 10.3389/fped.2022.784660

**Published:** 2022-02-25

**Authors:** Huijing Wang, Qingxiong Yu, Qingfeng Li, Zhichao Wang

**Affiliations:** Department of Plastic and Reconstructive Surgery, Shanghai Ninth People's Hospital, Shanghai Jiao Tong University School of Medicine, Shanghai, China

**Keywords:** large congenital melanocytic nevi, quality of life, Chinese, 7B rule, LCMN

## Abstract

**Background:**

Large congenital melanocytic nevus (LCMN) is a rare skin disease that deeply affects an individual's appearance, may influence patients' self-evaluation and social relationships, and further affects their quality of life (QoL). The Skindex-29 and 36-Item Short Form Survey (SF-36) are valid instruments used to evaluate QoL specifically. It is necessary to assess the QoL of patients with LCMN and summarize potentially impactful factors to help people understand LCMN patients and assist doctors in offering professional advice.

**Methods:**

Twenty-five patients were recruited from Shanghai Ninth People's Hospital from July 1st, 2019, to March 31st, 2021. Both males and females were included, and the age groups were divided into infants (0–6 y), children (7–12 y), teenagers (13–17 y) and youths (18–45 y). The Skindex-29 and SF-36 were applied as questionnaires for the assessment of QoL. Clinical information was acquired by physical examination.

**Results:**

QoL in patients with LCMN was diminished, especially in the emotional aspect. However, different genders, ages and distribution patterns of LCMN did not significantly influence QoL, but the patterns of “Bonce” and “Body” affected QoL the most and the severest. The results of Skindex-29 and SF-36 were consistent in that LCMN mainly reduced QoL from an emotional perspective.

**Conclusions:**

This research shows that LCMN has the strongest impact on patients' emotional wellbeing but weakly influences the whole fettle of QoL. The gender, age and distribution patterns of lesions all have no direct effect on QoL, although a larger proportion of LCMNs probably insinuates worse QoL. Even though patients with LCMN show better QoL than those with other visible skin conditions, their general mental health still requires ample attention from surroundings and professional doctors.

## Introduction

Large congenital melanocytic nevus (LCMN) is a rare skin disease that occurs in newborns at an incidence of 1/20,000 to 1/50,000 (1, 2). It presents as an obvious birthmark, deeply affecting an individual's appearance. In clinical practice, LCMN has numerous phenotypes, including the number of satellites, color heterogeneity, surface rugosity, extensive dermal or subcutaneous nodules and hypertrichosis (3). Some patients may state that they have a feeling of itch or fragility of skin. In association with congenital melanocytic nevus (CMN), there is a risk of developing melanoma and central nervous system abnormalities, and the risk is higher when CMN is larger (4, 5). However, in the largest LCMN cohort and longest follow-up study in Chinese patients, it was found that the malignancy rate of LCMN is at a very low level in China (6). Other available data also demonstrate that the risk of melanoma is overestimated, with 0.7% in CMN and up to 3.1% in LCMN (4, 7). In addition, it is already known that asymptomatic skin conditions caused by melanocytic cells proliferating or decreasing, such as vitiligo, result in adverse quality of life (QoL) (8). On the other hand, different phenotypes and distribution patterns of CMNs may cause disparities in patients' QoL as well as multiple kinds of treatments for the disease, yet 95 of 136 LCMN patients expressed that surgery did not have a positive effect on their QoL (9). Using common sense, people usually think that a smaller CMN leads to less impairment in QoL. However, early and recent studies have highlighted that the size or visibility of CMNs has no explicit correlation with impairment (10–12). Even though there are a few studies about the QoL of patients with CMN worldwide, none have been conducted in China thus far. The effect of culture, economy and society in different countries and regions on QoL should not be neglected. We speculated that there would be a profound and lasting influence on Chinese patients' self-evaluation and social relationships. Hence, it is valuable to estimate the distinctive QoL of patients with LCMN in China, and it is important for the public to pay close attention and show abundant goodwill to those who have rare diseases and are living in difficult circumstances.

There are many ways to assess QoL. The Skindex-29 is a technical and convenient instrument to evaluate the effects of skin diseases on patients' QoL. It is made up of 29 items concerning respondents' feelings over the past 4 weeks about the skin condition that have bothered them the most. It aims to assess three aspects of QoL: emotional states, burden of symptoms and social functioning. The 36-Item Short Form Survey (SF-36) is a reliable and valid instrument utilized for comprehensive routine monitoring and assessment of care outcomes in patients through eight subscales related to health: physical functional impairment (PF); social functional impairment (SF); limitations in usual role activities because of physical health problems (RP); bodily pain (BP); general mental health (MH); limitations in usual role activities because of emotional problems (RE) and vitality (VT); and general health perceptions (GH) (13, 14). Scores on the above eight subscales can be summed as two groups of composite scores: physical composite score (PCS), including PF, RP, BP and GH and mental composite score (MCS), including VT, SF, RE and MH (15).

Recently, a “7B” rule for classifying the distribution patterns of LCMN was proposed (6). It is meaningful to determine whether there is any connection between the “7B” rule and the QoL of patients with LCMN. Consequently, we explored the overall QoL of patients with LCMN in China and intended to summarize influential factors relating to QoL.

## Methods

### Patients

Forty LCMN patients who attended the Plastic and Reconstructive Surgery Clinic at Shanghai Ninth People's Hospital, Shanghai Jiao Tong University School of Medicine from July 1st, 2019 to March 31st, 2021 were recruited. LCMN was defined as its PAS being larger than 20 cm and smaller than 40 cm at its greatest diameter (3). Every recruited patient was diagnosed with LCMN by eligible clinical doctors. After excluding incompatible participants, 25 patients eventually met our inclusion criteria. These criteria included having been diagnosed LCMN and an exact description of the lesion's distribution pattern using the “7B” rule. Individuals who did not consent to participate in the research and who had incomplete questionnaires were excluded.

This study was approved by the institutional ethical committee of Shanghai Ninth People's Hospital, Shanghai Jiao Tong University School of Medicine (SH9H-2019-T163-2, September 30, 2019). Informed consent was obtained from all individual participants or their proxies.

### Questionnaires

The Mandarin version of the Skindex-29 translated by Mapi Research Trust and the RAND 36-Item Health Survey (Version 1.0) were adopted in this study. Every participant finished both the Skindex-29 and the SF-36. All answers on the Skindex-29 have five options: “never”, “rarely”, “sometimes”, “often” and “all the time”. These options align with the scores “0”, “25”, “50”, “75” and “100”, respectively. A higher score means a higher impact of skin disease on QoL. In simple terms, the higher the score, the poorer the QoL. Four degrees, “no”, “mild”, “moderate” and “severe”, are employed to describe QoL impairments (16, 17). Table 1 provides the cutoff score for every domain of the Skindex-29.

**Table 1:** Cut-off score for each domain of the Skindex-29.

	**No**	**Mild**	**Moderate**	**Severe**
Emotion	Score < 24	24 ≤ score < 35	35 ≤ score < 39	Score ≥ 39
Symptom	Score < 39	39 ≤ score < 42	42 ≤ score < 52	Score ≥ 52
Function	Score < 21	21 ≤ score < 32	32 ≤ score < 37	Score ≥ 37

The scoring method of the RAND 36-Item Health Survey 1.0 is distributed by the International Resource Center for Health Care Assessment (Boston, MA). In the SF-36, final scores are standardized on a scale of 0 to 100 in a certain way. Contrary to the Skindex-29, a higher score on this questionnaire indicates a better QoL.

Clinical information was collected, including patients' gender, age and the distribution patterns of lesions. The distribution pattern of LCMN referred to the “7B” rule, and every lesion was classified as “bonce”, “bolero”, “back”, “bathing trunk”, “breast/belly”, “body extremity” or “body” (6).

According to the Chinese common age classification standard, all patients were divided into four groups based on their age. The group called “infants” included patients from birth to 6 years old. The group called “children” included patients between 6 and 12 years old. The group called “teenagers” included patients between 13 and 17 years old. Finally, the group called “youth” included patients between 18 and 45 years old. Given that young children could not understand questions and answers well, guardians were their responsible proxy.

Both the Skindex-29 and the SF-36 were analyzed comprehensively to determine the association between various characteristics of patients with LCMN and their QoL. Meanwhile, the Skindex-29 and SF-36 were compared separately and integrated to further learn whether there was heterogeneity in the efficacy of the two questionnaires.

### Statistical Analysis

Descriptive statistics are presented as frequencies and percentages, whereas continuous variables are expressed as the mean and standard deviation (SD). Repeated measures ANOVA was used to detect any overall dissimilarity between related means. The Pearson correlation coefficient was employed to verify whether correlations existed. Student's *t* test was applied *post-hoc* to determine which paired comparisons were significantly different. The significance level was defined as a *P* value of <0.05, and the extreme significance level was <0.01. Statistical analysis was performed using SPSS® IBM® version 25 (SPSS Inc, Chicago, IL, USA).

We hypothesized that the QoL of LCMN patients would differ significantly based on gender, age and “7B” distribution patterns. Furthermore, it was necessary to identify the disparity of efficiency between the Skindex-29 and the SF-36 in evaluating the QoL of patients with LCMN.

## Results

### Clinical Information

In total, we received 41 answer sheets for the SF-36 and 40 for the Skindex-29. After excluding unqualified responses, 25 patients were enrolled, including 12 males and 13 females. There were 18 minors and five adults, who were further divided into four generation categories. As a result, there were 13 infants, four children, three teenagers and five youth. The participants' ages ranged from one to 34 years old, the mean age was 10.9 years old, and the standard deviation was 10.7. Eight people were investigated twice with the Skindex-29 over 20 months. Every patient's LCMN distribution pattern was described as the “7B” rule, with two being “bolero”, “back” and “bathing trunk” and one being “breast/Belly” and “body”. Fourteen lesions were classified as “bonce”, and three were classified as “body extremity” (Table 2).

**Table 2:** Clinical characteristics of patients with LCMN.

	**No. (%)**
**Gender**	
Male	12 (48)
Female	13 (52)
**Generation**	
Infants (0–6y)	13 (52)
Children (7–12y)	4 (16)
Teenagers (13–17y)	3 (12)
Youth (18–45y)	5 (20)
**“7B” Rule**	
Bonce	14 (56)
Bolero	2 (8)
Back	2 (8)
Bathing trunk	2 (8)
Breast/Belly	1 (4)
Body extremity	3 (12)
Body	1 (4)

### Outcomes of the Skindex-29

**Figure 1:**
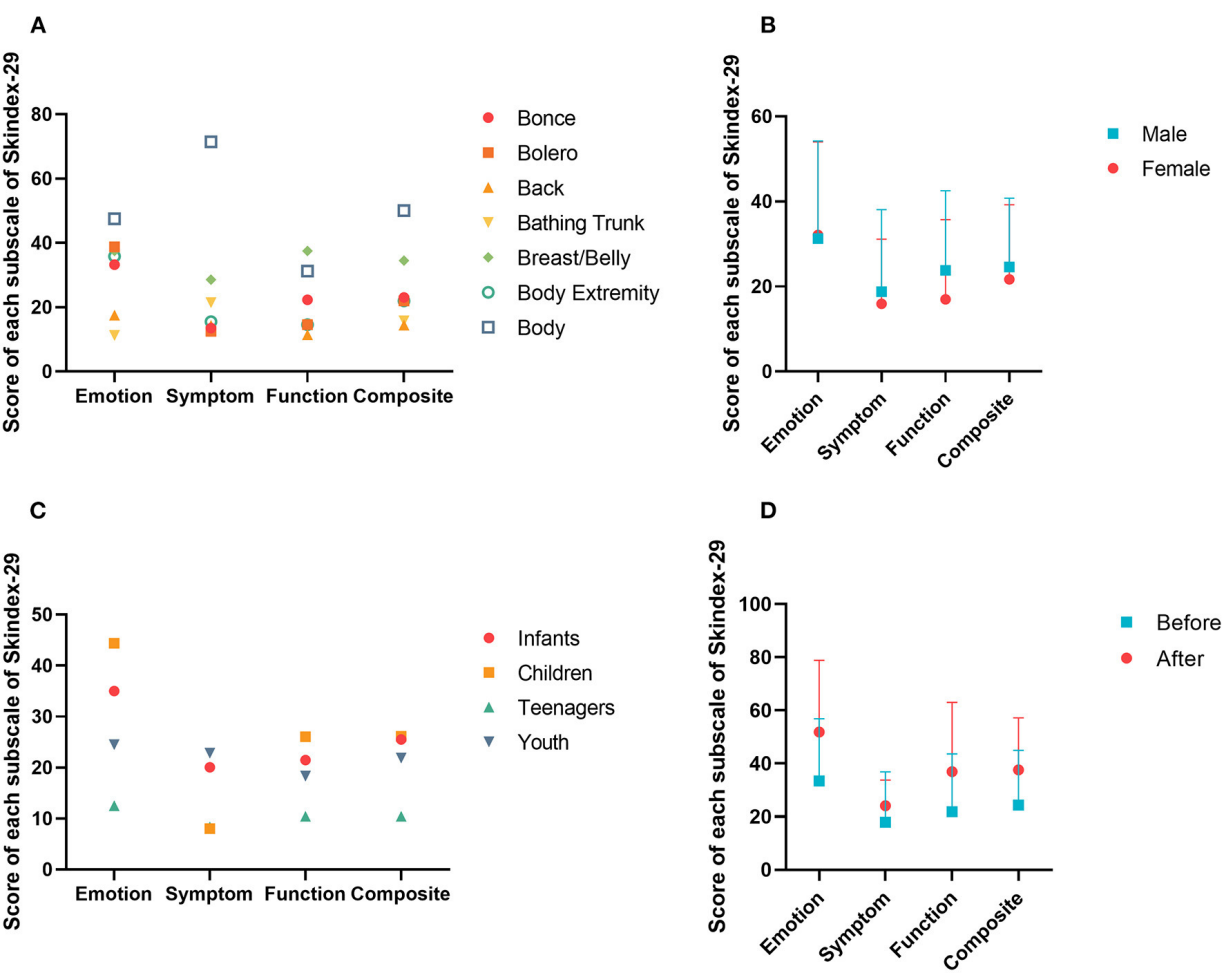
Score of each subscale in Skindex-29. **(A)** Scores of different distribution patterns of LCMN based on “7B” rule in four subscales. **(B)** Scores of different genders in four subscales. **(C)** Scores of different generations in four subscales. **(D)** Scores of different periods in four subscales.

All 25 patients completed the Skindex-29 entirely. The calculated scores of “emotion”, “function” and “symptom” of the Skindex-29 were normally distributed. These three items were pairwise correlated, and a *T* test showed that the mean score of “emotion” was significantly higher than “function” and that the mean score of “function” was significantly higher than “symptom” (Table 3). Different “7B” distribution patterns did not influence QoL in total or any separate aspect, except the distribution pattern of “body”, which was extremely significantly different from other distribution patterns in the domain of symptoms (Figure 1A). Likewise, the mean scores of “body” acquired on the “emotion” and “composite” subscales were both much higher than those of the other distribution patterns. The effects of gender and age were not significant for three aspects of QoL. However, the results directly showed that the mean scores of males were higher than those of females, except for the domain of emotion (Figure 1B). And the teenagers gained the lowest scores of all subscales of Skindex-29 (Figure 1C). Eight patients with LCMN completed the Skindex-29 once again 20 months after the first time. The result inferred that no difference existed, although participants had a higher mean score on each subscale after nearly 2 years (Figure 1D).

**Table 3:** Statistics of subscales of Skindex-29 and SF-36 in patients with LCMN.

	**Mean ±SD**	**Median**	**Quartiles**	
			**Q1**	**Q2**
**Skindex-29**				
Emotion	31.7 ± 22.0	35.0	15.0	47.5
Function	20.3 ± 18.7	16.7	4.2	31.3
Symptom	17.3 ± 17.0	14.3	3.6	21.4
Composite	23.1	23.4	10.3	32.2
**SF-36**				
PF	95.2 ± 11.2	100.0	95.0	100.0
BP	91.1 ± 22.0	100.0	100.0	100.0
RE	86.7 ± 30.4	100.0	100.0	100.0
GH	86.4 ± 15.8	95.0	75.0	100.0
SF	84.5 ± 16.6	87.5	75.0	100.0
RP	83.0 ± 31.2	100.0	75.0	100.0
VT	72.6 ± 16.7	75.0	60.0	85.0
MH	69.8 ± 18.2	72.0	75.0	100.0
PCS	88.9	92.5	87.5	98.8
MCS	78.4	82.6	70.5	89.1

*PF, physical functional impairment; BP, bodily pain; RE, limitations in usual role activities because of emotional problems; GH, general health perceptions; SF, social functional impairment; RP, limitations in usual role activities because of physical health problems; VT, vitality; MH, general mental health; PCS, physical composite score; MCS, mental composite score*.

**Figure 2:**
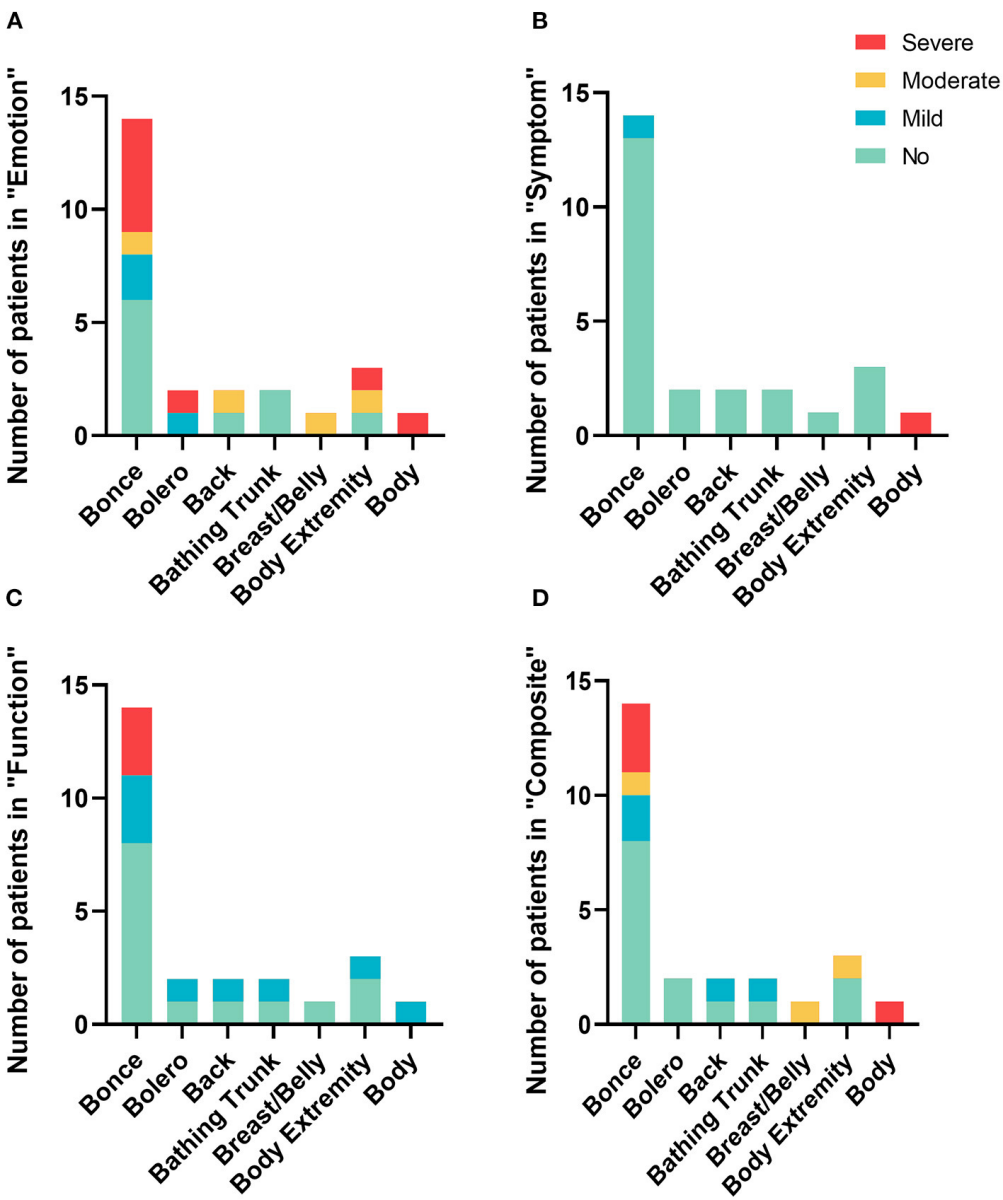
The number of patients with LCMN in different degrees of QoL among “7B” rule. **(A)** In the subscale of “Emotion”. **(B)** In the subscale of “Symptom”. **(C)** In the subscale of “Function”. **(D)** In the subscale of “Composite”.

Four grades were employed to describe the severity of QoL on each subscale of the Skindex-29. Specifically, they were “no influence”, “mild influence”, “moderate influence” and “severe influence”. Figure 2 shows the percentage of each grade in the categorized subgroups by the “7B” rule. In general, the outcome indicated that LCMN patients' emotional states were affected by skin disease the most, with 32.0% of patients feeling severe emotional impairment. Comparably, patients barely had a burden of clinical symptoms or any physical functions, except one individual whose LCMN distributed throughout the whole body who felt severely influenced in both the “emotion” and the “symptom” categories. Remarkably, the results reflected that cases of severe QoL generally occur in patients with “bonce” LCMN.

### Outcomes of the SF-36

**Figure 3:**
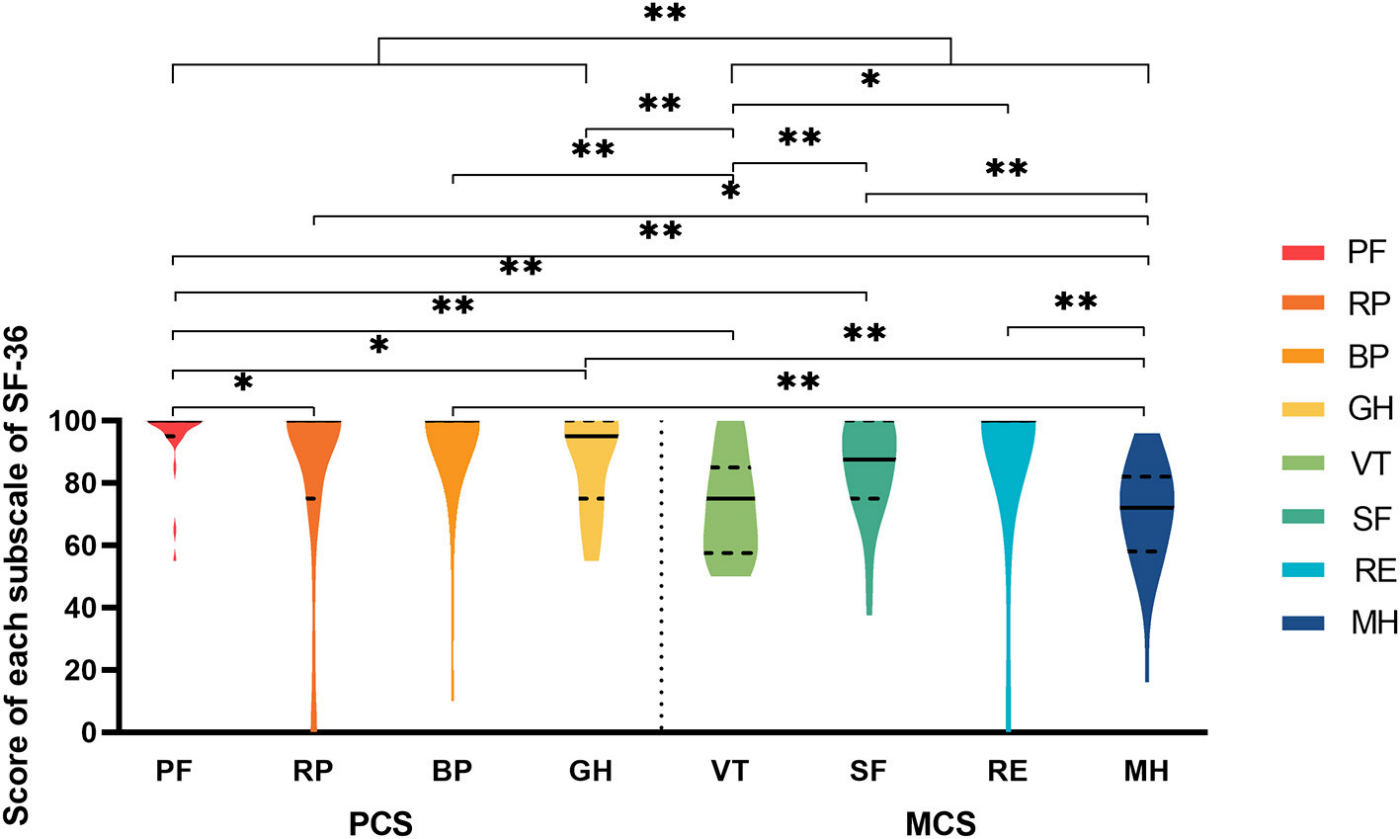
The violin plot of eight subscales in SF-36. PF, physical functional impairment; RP, physically limited usual role activities; BP, bodily pain; GH, general health; VT, vitality; SF, social functional impairment; RE, emotionally limited usual role activities; MH, mental health; PCS, physical composite score; MCS, mental composite score. *significant difference; **extreme significant difference.

Figure 3 shows the results of the SF-36, and there were extremely significant differences on the eight subscales. Notably, an extremely significant difference existed between the PCS and MCS groups. There were conspicuous disparities between PF and the other subscales, except RE and BP. In addition, the results showed that PF not only had the highest mean score but was also the most concentrated subscale, with a mean ± SD equal to 95.2 ± 11.2. The median and mode were both 100.0, the minimum was 55.0, the first quartile was 95.0 and the third quartile was 100.0. In contrast, only RP had the minimum score of zero among the eight subscales, but the mean ± SD was 83.0 ± 31.2, and the median was 100.0, which was not the lowest. On average, scores of MH were the lowest and the MCS scores were distributed beneath the PCS on the Y-axis.

Taking the “7B” rule into account, the pair of “bonce” and “back” as well as the pair of “bonce” and “extremity” showed extremely significant differences in QoL on the VT subscale. In addition, “bathing trunk” and “boreo” as well as “bathing trunk” and “extremity” also showed extremely significant differences in QoL on the GH subscale. Five patients reanswered the SF-36 after a period of 20 months. The influence of time was negative, which meant that the fettles of patients' QoL were stable with increasing age. This result was consistent with the conclusion from the Skindex-29.

## Discussion

It is widely acknowledged that there is a risk that CMN will transform into malignant melanoma or involve central nervous system abnormalities, which will cause a major threat to life. Despite numerous congenital or acquired birthmarks that affect patients' appearances being studied in detail, only a few studies have focused on CMN, not LCMN. Because patients with LCMN suffer constantly in their lives and pressures come from both society and themselves, we performed research about their QoL using the Skindex-29 and SF-36. This is the first study in China, even in Asia, that pays close attention to LCMN, a rare disease, and cares about these patients' emotional and physical wellbeing.

This research was conducted in a single center and involved the recruitment of 25 patients; the numbers of males and females were almost the same. However, a recent study found that LCMN was more prevalent in females (9). This is partly due to the rarity of LCMN. Generation categories referred to the Chinese common standard of age classification, which was the most appropriate choice for research in China. The results demonstrated that the majority of individuals were at the infant stage, which was reasonable and predictable. As a congenital disease, LCMN exists before the baby is born. Once the birthmark is noticed, anxious parents choose to remove it from their children as soon as possible to reduce its impact on their maturation. Thus, newborns are brought immediately to doctors in early infancy. Eighty percent of patients were under 18 years old, which suggests that patients and their families are always eager to solve skin problems before adulthood to prevent harmful influences on patients' lives if lesions have not been excised successfully at infancy. The statistical results of the “7B” rule verified the latest finding that “bonce” is the most frequent pattern of LCMN (6).

According to the Skindex-29, only the patient with LCMN of the “body” pattern conveyed much lower QoL than others, which indicates that the larger cover area of LCMN possibly causes more negative effects on all aspects of life. In particular, the reduced QoL mainly results from symptoms of LCMN, such as itch and pain. However, we cannot deny the fact that the number of patients with “body” LCMN was too small to obtain accurate statistical results in the study. On the other hand, the number of patients with LCMN distributed as “bonce” and “body” who felt severely unsatisfactory in terms of their QoL was much greater than that of other distribution patterns, perhaps because their skin lesions were either the most obvious or had the largest area.

Unlike previous studies of some dermatological diseases, such as port-wine stains and cutaneous lupus erythematosus, young age was correlated with diminished QoL (8, 18), age has less to do with QoL in patients with LCMN (10, 12). In our analysis, patients with LCMN in every age group presented no significant difference in QoL, which is consistent with conclusions drawn from studies about other districts and races. However, the lowest scores reflected in teenagers inferred that individuals in puberty experience much more sensitivity which should be paid sufficient attentions to. After 20 months of follow-up, the patients' QoL tended to decrease with increasing age, but this trend was not meaningful in the statistical analysis. A likely crucial reason is that the follow-up period was too short, and the number of participants for comparison was inadequate. Although 20 months had passed, few children with a low mental and cognitive level were not able to be aware of their skin disease. Therefore, there is no urgent need for young people to eliminate lesions on their skin if the time for operations or other treatments is inappropriate.

The SF-36 and Skindex-29 both are composed of concepts related to emotional state, physical function and clinical symptoms. However, the SF-36 has more detailed subscales and focuses on generic health-related QoL affected by diverse elements, whereas the Skindex-29 merely focuses on abnormal situations caused by skin diseases. As mentioned above, PCS and MCS had significantly different impacts on QoL, and the scores of the former were higher than those of the latter on average, which implies that mental and emotional factors cause primary impairment in QoL and that patients with LCMN may generally suffer more mental problems. For instance, losing interest and vitality in daily social interactions and working or being afraid to build intimate relationships with others are common. Over time, the loneliness and stress resulting from social isolation become overwhelming, so numerous patients with physical disease end up with terrible mental illnesses, such as depression and anxiety. In an early study, Picardi et al. pointed out a prevalence rate of more than 30% of psychiatric illnesses in patients with various skin diseases (19).

Hagen et al. compared port-wine stains with 13 other skin diseases, including dermatomyositis, psoriasis and rosacea, applying the Skindex-29, and the sample sizes of those studies were quite similar to ours (8, 15, 18, 20–24). Scores on all four subscales of LCMN were very low, with a rank of 13th on all 16 skin conditions, which demonstrates that LCMN affects patients' QoL much less than other visible skin conditions.

Furthermore, we associated the “7B” rule and other factors with patients' QoL and analyzed the possible correlation. Although we have drawn a couple of meaningful conclusions, several limitations still exist that cannot be ignored. First, the total number of patients recruited in this study was small and the follow-up time is short not only due to the rarity of LCMN but also because the recruitment source was a single hospital, which seriously limited the size of the sample and easily caused selection bias. Second, individuals of pediatric age lack sufficient knowledge and cognition to read and understand questions and answers in questionnaires, resulting in inaccurate and imprecise outcomes. To our knowledge, there are some instruments created for individuals of that age specifically. For example, the Dermatology Life Quality Index (DLQI) was developed for adolescents (25) and the cartoon version of the DLQI was created for children beyond 4 years old (26). However, the questionnaires used in our research not only evaluated children's QoL but also reflected the perspective of parents about their children and LCMN. On the one hand, the results showed poor emotional circumstances in most patients. On the other hand, it highlighted the anxiety and unstable emotional states of their parents. Therefore, the disease actually affects everyone's QoL in the family and creates a burden for the whole family. Further research is still needed to re-evaluate the QoL of these patients with suitable and qualified procedures. Third, information was collected insufficiently. For this reason, we might have missed many elements that have effects on QoL, such as multiple kinds of treatments and comorbidities.

In conclusion, our research shows that LCMN has the strongest impact on the patient's emotional wellbeing but weakly influences the whole fettle of QoL. The gender, age and distribution patterns of lesions all have no direct effect on QoL, although a larger proportion of LCMNs probably insinuates worse QoL, as the patterns of “bonce” and “body” affect QoL the most and the severest. Although patients with LCMN show higher QoL than individuals with other similar skin conditions, their general mental health still requires extra ample attention. The family and surroundings should offer more kindness and comprehension to support patients positively. Doctors are also supposed to provide professional medical advice opportunely when patients first seek treatment. Guidance and management of patients with LCMN need to be improved, and timely referrals to psychosocial specialists are essential.

## Data Availability Statement

The original contributions presented in the study are included in the article/supplementary material, further inquiries can be directed to the corresponding authors.

## Ethics Statement

The studies involving human participants were reviewed and approved by Institutional Ethical Committee of Shanghai Ninth People's Hospital, Shanghai Jiao Tong University School of Medicine (SH9H-2019-T163-2, September 30, 2019). Written informed consent to participate in this study was provided by the participants' legal guardian/next of kin.

## Author Contributions

QL and ZW contributed to the conception, design of the work, and defined the intellectual content. HW, QY, and ZW did literature search. HW and QY finished clinical study, data acquisition, data analysis, statistical analysis, and manuscript preparation. ZW edited the manuscript. QL reviewed the manuscript. All authors substantively revised the work and have approved the submitted version.

## Funding

This work was supported by grants from National Natural Science Foundation of China (82102344; 82172228); Shanghai Rising Star Program supported by Science and Technology Commission of Shanghai Municipality (20QA1405600); Science and Technology Commission of Shanghai Municipality (19JC1413); Chenguang Program supported by Shanghai Education Development Foundation (SHEDF) (19CG18); Shanghai Municipal Key Clinical Specialty (shslczdzk00901); Innovative research team of high-level local universities in Shanghai (SSMU-ZDCX20180700).

## Conflict of Interest

The authors declare that the research was conducted in the absence of any commercial or financial relationships that could be construed as a potential conflict of interest.

## Publisher's Note

All claims expressed in this article are solely those of the authors and do not necessarily represent those of their affiliated organizations, or those of the publisher, the editors and the reviewers. Any product that may be evaluated in this article, or claim that may be made by its manufacturer, is not guaranteed or endorsed by the publisher.
